# Closures of Cavities in Bones

**Published:** 1919

**Authors:** 


***Closures of Cavities in Bones.*** By Lieutenant-Colonel PERCY SARGENT. *The Lancet,* December 14, 1918.

*Processes of Repair in Bone.* The fact that the walls are not collapsible hinders considerably the healing of cavities in bones. Cancellous bone is more adapted for recovery than compact bone. Two processes combine to bring about the coalescence of the granulations covering the wall of a cavity : (1) the falling together of the adjacent structures; and (2) the process of contraction. In the case of a bone cavity both these methods would be so slow as to need longer than the life-time of the patient.

*Methods of Filling Cavities in Bone.* This problem has been attacked in different ways, and since 1886 the following processes have been resorted to successively.

(1) An Esmarck's bandage having been applied to the limb, the cavity was cleaned very carefully, and the wound closed. On removal of the bandage, the cavity filled with blood which became organized into, or replaced by fibrous tissue.

(2) Pieces of sponge had been packed into the cavity to form a framework for the growth of granulations.

(3) One of the bony walls of the cavity was removed and flaps of skin were secured in position into the cavity by means of nails.

(4) The cavity, rendered as aseptic as possible, was firmly packed with clips of decalcified bone, prepared by soaking in 500 sublimate alcohol.

(5) A method of plugging bone cavities with a paste compounded of iodoform, spermaceti and oil of sesame was described by Morhof and Jones in 1905. Both these last methods and the first one require conditions rarely attainable, since the walls of the cavity should contain no diseased tissue and should be perfectly aseptic.

(6) Hollander carefully cleansed and prepared the cavity and filled it up by pouring melted human fat into it.

(7) Broca's radical operation aimed at the obliteration of the cavity by the falling in of the soft parts. The bone was freely exposed through an incision, and the cavity converted into an open trough by removal of one of its walls. Any sequestra and all diseased tissue were carefully removed, and the overlying parts were then encouraged to sink into and gradually to obliterate the resultant cavity.

*Technic of Modification of Broca's Operation.* The method which the author advocates might be called that of continuous muscle-grafting.

No tourniquet was employed, for the subsequent oozing was a great drawback. It was easier to judge of the condition of a bone which bled under the curette than of one temporarily deprived of its blood supply, and sloughing might develop in an injured skin totally deprived of blood-supply for an hour or more. Hemorrhage was easily controlled by frequent application of large pieces of gauze soaked with very hot saline. A sufficiently thorough exposure of the bone, both above and below the cavity, must be secured. Wide excision of the wall of the sinus leading to the cavity was the first operatory step. The periosteum was next incised to the extent of the whole length of the wound, and stripped from the bone so as to bare it completely. Lane's bone levers rather than ordinary retractors were then inserted between the periosteum and the bone. It was most important to pack the bone off by means of gauze pads to prevent fragments of bone getting lost among the soft parts. The cavity in the bone was fully opened up by means of a chisel and mallet and all granulation tissue and dead or carious bone removed. The cavity was next washed out with hot saline solution and plugged tightly with gauze.

The second stage of the operation was not commenced until all soiled packing had been taken away, the towels changed, the instruments re-sterilized and the surgeon's and assistant's gloves changed. The best method of filling the cavity with muscle was to fashion from the most conveniently situated mass of muscle, a thick, broad, pedicled flap of approximately the same size as the cavity. It is most important to secure an adequate bloodsupply to the transplanted muscle. The muscle graft was pushed into the cavity and kept in position by a few stiches of catgut. The skin was loosely sutured and a certain number of drains were placed in order to provide the escape of the exudate. Finally the limb was splinted in such a way as to relax the parent muscle.

Healing by first intention occurred in a few cases, but a considerable reaction, lasting for several days, usually took place.

*Advantages of the Muscle-graft Method.* The principal advantage over Broca's method was that less bone need be removed, and that the whole cavity need not be converted into an open gutter. The filling-up of the space by means of muscle-grafting was immediate and complete; the blood vessels of both muscle and bone coalesced, bringing forth an additional blood-supply to the bone. No evidence was obtained of bone-formation in the muscle, but this was of no importance from the standpoint of strength, for, as the patient made use of the limb, and so subjected the bone to varied strains, compensatory overgrowth occurred.

